# Fluoride Ionic Liquids in Salts of Ethylmethylimidazolium and Substituted Cyclopropenium Cation Families

**DOI:** 10.3389/fchem.2018.00603

**Published:** 2018-12-10

**Authors:** Owen J. Curnow, Douglas R. MacFarlane, Kelvin J. Walst

**Affiliations:** ^1^School of Physical and Chemical Sciences, University of Canterbury, Christchurch, New Zealand; ^2^ARC Centre of Excellence for Electromaterials Science, Monash University, Melbourne, VIC, Australia

**Keywords:** ionic liquids, fluoride, imidazolium, cyclopropenium, salts, solvate

## Abstract

A series of solvent-stabilized ionic liquid fluorides were prepared, [EMIM]F.nCH_3_COOH *n* = 1. 0, 1.6, 2.1, 2.4, and 3.2, either via exchange from the chloride salt using KF or AgF, or by neutralization of the hydroxide salt using HF. Azeotrope drying was used to remove water. Their viscosity, conductivity and density properties were determined. A diethanol solvate of the triaminocyclopropenium salt [C_3_(NPr_2_)_3_]F was found to be stable and its viscosity, conductivity and density properties were also determined. The monoethanol solvate, however, was found to be unstable with trace water present. Intramolecular stabilization of fluoride was achieved by using OH functionalized cations: [C_3_(NEt_2_)_2_N(CH_2_CH_2_OH)_2_]F, [C_3_(N(CH_2_CH_2_OH)_2_)_3_]F, and [Me_3_NCH_2_CH_2_OH]F.H_2_O, The first of these is an ionic liquid at ambient temperature and has a TGA mass loss onset at 175°C, indicating a useful range of liquid state stability.

## Introduction

Research in Ionic Liquids (ILs) has explored a vast variety of anion types, revealing a wide range of properties and applications. Halide anion based ILs were some of the first known, especially those based on iodide due to the generally lower melting points with increasing size and polarizability in Group 17. Generally, fluoride salts are high melting and therefore have attracted little recent attention in this field. On the other hand, fluoride anions have the potential to make ionic liquids with interesting properties due to the reactivity of fluoride (Maiti et al., 2008; Mallik and Siepmann, 2010). Indeed, fluoride salts, such as tetrabutylammonium fluoride (TBAF), have many uses as fluorinating reagents and for the removal of silicon-based protecting groups. The more “naked” (higher donor ability) the fluoride anion is, the higher the reactivity (Christie and Jenkins, 2003). However, this reactivity causes difficulties with organic fluoride salts often being difficult to use, either because they become unstable due to the reactive fluoride (Sun and DiMagno, 2005) or they are insoluble in aprotic solvents.

Triaminocyclopropenium (TAC) cations have been considered as counter ions to fluoride for two reasons: firstly, there are no acidic protons, and secondly, TAC cations have a high energy HOMO that leads to weak cation-anion interactions and good ionic liquid properties (Weiss et al., 1995a,b; Butchard et al., 2006; Curnow et al., 2011, 2012; Wallace et al., 2015). Cations with acidic protons will easily be deprotonated by fluoride, which becomes a much stronger base as it becomes less solvated. This is predicted to occur in imidazolium fluoride salts: *ab initio* calculations on 1-ethyl-3-methylimidazolium fluoride, [EMIM]F, show that covalent bonding between the fluorine atom and a proton extracted from the 2 position of the imdazolium cation is preferred over the ionic form. The result is HF and a stable Arduengo carbene (Turner et al., 2003; Maiti et al., 2008). As a result, imidazolium-based ionic liquid fluorides have only been reported with solvated fluoride anions (Swatloski et al., 2003; Maiti et al., 2008). TAC cations might be more resistant to Hofmann elimination, which causes the decomposition of tetraalkylammonium fluorides. On the other hand, TAC cations are sensitive to strong nucleophiles, such as hydroxide, and possibly fluoride itself. For this reason, even if triaminocyclopropenium salts proved to be stable to fluoride, any kind of naked fluoride ionic liquid would be highly moisture sensitive.

A number of solvate salts of fluorides are known, for example the *t*-butanol solvate of tetrabutylammonium fluoride, TBAF(^t^BuOH)_4_, which is a fluoride solvate that has been investigated as an anhydrous source of fluoride (Kim et al., 2008). However, due to the symmetric shape of the ions and *t*-butanol, and the non-dispersed charges, it is a solid.

Larson (Larson and McMahon, 1983) carried out gas phase measurements of the binding energy of fluoride with a variety of Brönsted and Lewis acids. The binding energy of the ethanol to fluoride hydrogen bond was found to be 132 kJ mol^−1^, a strong hydrogen bond that is not much weaker than that measured for hydrogen fluoride to fluoride (bifluoride) at 161 kJ mol^−1^. Other measurements have suggested this was low and found a bond energy of 191 kJ mol^−1^ (Wenthold and Squires, 1995). The strong hydrogen bond suggests that the alcohol solvate can be thought of as a discrete species. Fluorohydrogenate ([F(HF)_*x*_]^−^) anions in ionic liquids are considered as a discrete species, and have received considerable attention in the literature due to high conductivities and low viscosities (Hagiwara et al., 1999, 2003).

In this paper, we report on TAC fluoride salts with inter- and intra-molecular alcohol solvate interactions. However, the solvate molecules do not need to be limited to alcohols, they just need to be good hydrogen bond donors. So, prompted by earlier work on dimeric and higher order oligomeric acetic acid anions (Johansson et al., 2008), acetic acid was also investigated as a solvate molecule with imidazolium fluoride salts. Both the [AcOHF]^−^ and [(AcOH)_2_F]^−^ species, shown below, have been observed previously by NMR at sub-ambient temperature (Golubev et al., 2004).


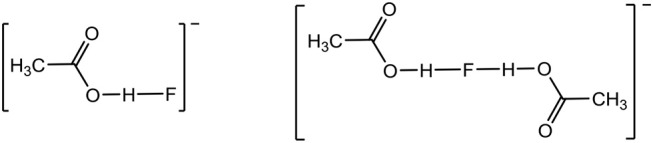


## Experimental

### General Procedures

All operations were performed using standard Schlenk techniques with a dinitrogen atmosphere in order to reduce exposure to water. ^1^H-, ^13^C{^1^H}-NMR spectra were collected on a Varian Unity-300 operating at 300 and 75 MHz, respectively, or on a Varian INOVA-500 operating at 500 and 126 MHz, respectively, in CDCl_3_ or D_2_O, referenced to residual solvent peaks or TMS. ^19^F-NMR spectra were collected on a Varian Unity-300 operating at 282 MHz, in CDCl_3_ or D_2_O, referenced to C_6_H_5_F or CFCl_3_. Electrospray mass spectrometry was carried out on a Micromass LCT, with samples dissolved in acetonitrile. Water contents were determined by Karl Fischer titration using a Metrohm 831 KF coulometer. Chloride contents were determined using a AutolabEco Chemie, with associated GPES software, under a dinitrogen atmosphere. The electrodes were either a glassy carbon (3 mm diameter) or platinum (1 mm diameter) working electrode, a platinum wire counter electrode and a silver reference electrode. Fluoride, Ag^+^ and K^+^ were determined similarly using ISEs. Mircoanalysis was performed by Campbell Microanalytical Laboratory, Dunedin. [C_3_(NPr_2_)_3_]Cl (Curnow et al., 2011) and (Et_2_N)_2_C_3_O (Curnow et al., 2012) were prepared by previously-published methods. Other reagents were used as obtained commercially.

Differential Scanning Calorimetry (DSC) was carried out on a TA Q100 DSC and a Perkin-Elmer DSC 8000. The DSC 8000 was calibrated with indium (156.60°C) and cyclohexane (−87.0 and 6.5°C). Three heating and cooling cycles were carried out with data collected from a repeatable second cycle. A scan rate of 10°C min^−1^ was used, over a temperature range of at least −100 to 50°C, with a greater range used as required. Transition temperatures are reported as the onset temperature. Enthalpy for a transition was found by integrating the peak area, with entropy then calculated using ΔS = ΔH/T. Sample size was ~5–10 mg. Density measurements were carried out on an Anton Parr DMA 5000 instrument, an oscillating U-tube density meter, from 20 to 90°C in 10°C steps. All imidazolium fluoride acetic acid mixtures were measured on an Anton Parr AMVn automated microviscometer. The other samples were measured on a Brookfield DV-II+ Pro cone and plate viscometer. Samples that were measured using the cone and plate viscometer were examined for non-Newtonian behavior, although this was only over a limited shear rate range. All samples were measured from 20 (or above the melting point if solid) to 80 or 90°C, and performed sealed or under a dinitrogen gas flow. Conductivities were measured by AC impedance spectroscopy on a Solatron SI 1296 frequency response analyser, at ranges up to 0.01 Hz to 10 MHz. Measurements were carried out with a dip cell probe containing two platinum wires covered in glass. The resistance was identified using a Nyquist Plot, and conductivity then calculated using κ = *l*/*AR*, where *l*/*A* is the cell constant, which was determined using 0.01 mol L^−1^ KCl solution at 25°C. All samples were measured from 20 (or above the melting point if solid) to 80 or 90°C, and performed sealed or under a dinitrogen gas flow. Thermal Gravimetric Analysis (TGA) was carried out on a TA Q600 SDT (simultaneous DSC-TGA), using platinum pans. Temperature calibration carried out using zinc (419.6°C), and heat flow using a sapphire standard. Pans were cleaned after each TGA experiment by heating at 500°C under an air atmosphere, and if required cleaned with nitric acid. Sample size was ~10 mg.

### Syntheses

Tris(diethanolamino)cyclopropenium chloride, [C_3_(N(C_2_H_4_OH)_2_)_3_]Cl (**1**)

Diethanolamine, HN(C_2_H_4_OH)_2_, (97.1 g, 0.925 mol) was mixed with CH_2_Cl_2_ (250 mL), and the suspension was cooled to 0°C. Pentachlorocyclopropane, C_3_Cl_5_H (24.7 g, 0.115 mol) was added dropwise and the solution stirred at ambient temperature overnight. The mixture was then heated to reflux for 24 h. Dichloromethane was then removed *in vacuo*. The mixture was dissolved into ethanol (150 mL) and molecular sieves (13X, 10 Å pore size) were added slowly until the solution was completely absorbed into the sieves. The crude product was extracted from the molecular sieves by soaking in ethanol (100 mL for 3 h followed by 100 mL for 19 h). This was repeated using methanol (2 × 100 mL), but with overnight soakings. Alcohol from the four fractions was removed *in vacuo*, yielding a pale yellow viscous liquid (24 g). All fractions were ~90% tris(diethanolamino)cyclopropenium chloride, with 10% [H_2_N(C_2_H_4_OH)_2_]Cl, as determined by NMR. The molecular sieves were washed with excess water to remove [H_2_N(C_2_H_4_OH)_2_]Cl, and the process was repeated to ultimately yield a pale yellow, extremely viscous liquid (7.0 g, 16%). ^1^H NMR (D_2_O, 300MHz): δ 3.81 (t, *J* = 5.3 Hz, 12H, NCH_2_), 3.61 ppm (t, *J* = 5.3 Hz, 12H, NCH_2_C*H*_2_). ES^+^ m/z 348.2158 (100%, M^+^).

Bis(diethylamino)diethanolaminocyclopropenium iodide, [C_3_(NEt_2_)_2_N(C_2_H_4_OH)_2_]I (**2**)

The cyclopropenone (Et_2_N)_2_C_3_O (7.68 g, 39.2 mmol) was dried using ethanol azeotropes (5 × 50 mL). Iodoethane (97.5 g, 625 mmol) was added and the solution heated to reflux for 18 h. After cooling to ambient temperature, HN(C_2_H_4_OH)_2_ (8.44 g, 80.3 mmol) was added with stirring for 48 h. Excess iodoethane was removed *in vacuo* and water (100 mL) added. Cyclopropenone was extracted with dichloromethane (100 mL and 50 mL). The product was then back-extracted with water from this dichloromethane solution (2 × 100 mL) and recombined with the first water layer. The final product was then extracted with CH_2_Cl_2_ (3 × 100 mL) to yield a yellow liquid (3.50 g, 22%). ^1^H NMR (300 MHz, D_2_O): 3.73 (t, *J* = 5.3 Hz, 4H, NCH_2_C*H*_2_OH), 3.47 (t, *J* = 5.3 Hz, 4H, NC*H*_2_CH_2_OH), 3.37 (q, *J* = 7.0 Hz, 8H, NC*H*_2_CH_3_), 1.17 (t, *J* = 7.2 Hz, 12H, NCH_2_C*H*_3_). ^13^C NMR (75 MHz, D_2_O, methanol reference): 117.89 (C_1_), 117.26 (C_2_), 59.46 (NCH_2_*C*H_2_OH), 54.38 (N*C*H_2_CH_2_OH), 47.20 (N*C*H_2_CH_3_), 13.98 (NCH_2_*C*H_3_).

Tris(diethanolamino)cyclopropenium fluoride, [C_3_(N(C_2_H_4_OH)_2_)_3_]F (**3**) via HF

Tris(diethanolamino)cyclopropenium chloride (**1**) (7.0 g, 19 mmol) was dissolved in water (250 mL) and Ag_2_O (7.0 g, 30 mmol) was added and stirred for 3 h in a blacked out flask. Water was partially (200 mL) removed *in vacuo*. AgCl was removed by filtration through Celite before the solution was neutralized to pH 7.5 with aqueous HF. Complete drying was achieved with additions (3 × 150 mL, 3 × 50 mL) of ethanol and isopropanol and removal of the solvent *in vacuo*. This was followed by extended drying under high vacuum with stirring and mild heat (40°C) to give a colorless solid (5.63 g, 84%). ^1^H NMR as for [C_3_(N(C_2_H_4_OH)_2_)_3_]Cl. ^19^F NMR (282 MHz, D_2_O, C_6_H_5_F): 122.05 (s). ^13^C NMR (75 MHz, D_2_O, acetonitrile reference): 118.18 (C_3_), 59.38 (NCH_2_*C*H_2_OH), 54.29 (N*C*H_2_CH_2_OH).

Tris(diethanolamino)cyclopropenium fluoride, [C_3_(N(C_2_H_4_OH)_2_)_3_]F (**3**) via AgF

Compound **1**, [C_3_(C_2_H_4_OH)_3_]Cl (3.0 g, 7.8 mmol) was dissolved in water (200 mL) and AgF (1.30 g, 10.2 mmol) added with stirring. AgCl was removed by filtration through Celite. Excess AgF was removed by removal of solvent *in vacuo*, followed by the addition of alcohol solvent (50 mL, 5 × ethanol then 5 × isopropanol), after each addition the solution was filtered through Celite and the solvent removed *in vacuo*. This yielded a light yellow, extremely viscous liquid (2.3 g, 80%). Found: C, 48.82; H, 8.09; N, 11.06%. Calc. for C_15_H_30_N_3_O_6_F: C, 49.04; H, 8.23; N, 11.44%. Cl^−^ content: 1,600 ppm.

Bis(diethylamino)diethanolaminocyclopropenium fluoride diethanol, [C_3_(NEt_2_)_2_N(C_2_H_4_OH)_2_]F (**4**)

Compound **2**, [C_3_(NEt_2_)_2_N(C_2_H_4_OH)_2_]I (3.50 g, 8.52 mmol) was stirred in water (250 mL) in a blacked-out flask to which Ag_2_O (3.0 g, 13 mmol) was added with stirring for 3 h. AgI was removed by filtration through Celite before the solution was neutralized to pH 7.5 with aqueous HF. The solution was reduced in volume to 20 mL *in vacuo*, with drying being completed with ethanol azeotrope drying (3 × 200 mL). This yielded the product as a viscous yellow liquid (2.5 g, 96%). ^19^F NMR (282 MHz, D_2_O, C_6_H_5_F reference): 121.94 (s).

Tris(dipropylamino)cyclopropenium fluoride diethanol, [C_3_(NPr_2_)_3_]F.1.9EtOH (**5**.1.9EtOH)

Tris(dipropylamino)cyclopropenium chloride, [C_3_(NPr_2_)_3_]Cl (10.6 g, 28.5 mmol) was stirred in water (450 mL), in a blacked-out flask, to which Ag_2_O (6.70 g, 28.9 mmol) was added and then stirred for 3 h. AgCl was removed by filtering through Celite, before the solution was neutralized to pH 7.1 with aqueous HF. The solution was reduced in volume to 50 mL *in vacuo*, with drying being completed with ethanol azeotrope drying (3 × 500 mL), which yielded the product as an orange liquid (12.4 g, 97%). ^1^H NMR (300 MHz, CDCl_3_): 3.68 (q, *J* = 7.0 Hz, 3.81H, CH_3_C*H*_2_OH), 3.27 (t, *J* = 7.9 Hz, 12H, NC*H*_2_CH_2_CH_3_), 1.67 (m, 12H, NCH_2_C*H*_2_CH_3_), 1.21 (t, *J* = 7.0 Hz, 5.00H, C*H*_3_CH_2_OH), 0.94 (t, *J* = 7.3 Hz, 18H, NCH_2_CH_2_C*H*_3_). ^19^F NMR (282 MHz, D_2_O, C_6_H_5_F): 122.03 (s). Found: C, 61.95; H, 12.48; N, 8.77%. Calc. for C_21_H_42_N_3_F.1.9EtOH.2.09H_2_O: C, 61.95; H, 12.07; N, 8.74%. H_2_O content: 2,200 ppm. F^−^ content: 44,300 ppm, calculated: 42,900 ppm.

1-Ethyl-3-methylimidazolium fluoride diethanol, [EMIM]F.1.8EtOH (**6**.1.8EtOH)

To [EMIM]Cl (33.0 g, 225 mmol) in ethanol (500 mL) was added KF (30 g, 520 mmol) suspended in ethanol (250 mL). After stirring for 1 h, the solid was removed by filtration. Two further additions of KF (35 and 25 g), with stirring (1 h) and removal, were made. The solvent was reduced in volume *in vacuo*, and acetonitrile (250 mL) was added and the solution cooled. A precipitate formed, and was filtered off. Ethanol was added to the filtrate, and excess solvent removed *in vacuo*. Cl^−^ content: 3,120 ppm. To the [EMIM]F ethanol solvate (52 g) in water (200 mL) was added AgF (550 mg, 4.34 mmol) in water (100 mL). AgCl precipitate was removed by filtration. The solvent volume was reduced *in vacuo* with drying completed using ethanol azeotropes. The product was a pale yellow liquid (41.9 g, 87%) with 1.83 ethanol solvate molecules per fluoride. ^1^H NMR (400 MHz, D_2_O): 8.63 (s, 1H, C*H*), 7.40 (d, *J* = 2.0 Hz, 1H, CH), 7.33 (d, *J* = 2.0 Hz, 1H, CH), 4.14 (q, *J* = 7.5 Hz, 2H, C*H*_2_CH_3_), 3.80 (s, 3H, C*H*_3_), 3.56 (q, *J* = 7.3 Hz, 3.6H, CH_3_C*H*_2_OH), 1.41 (t, *J* = 7.5 Hz, 3H, CH_2_C*H*_3_), 1.09 (t, *J* = 7.3 Hz, 5.4H, C*H*_3_CH_2_OH). ^19^F NMR (377 MHz, D_2_O, CFCl_3_): −121.22 (s). Cl^−^ content: 69 ppm. K^+^ content: < 100 ppm. Ag^+^ content: 821 ppm.

1-Ethyl-3-methylimidazolium fluoride acetic acid, [EMIM]F.1.0AcOH (**6**.1.0AcOH)

Acetic acid (1.77 g, 29.4 mmol) was added to [EMIM]F.1.8EtOH (6.31 g, 29.4 mmol). The mixture was dried with isopropanol (2 × 50 mL) and ethanol (50 mL) azeotropes. Additional acetic acid (0.111 g, 1.85 mmol) was then added. The product was a pale yellow liquid (5.04 g, 90%). ^1^H NMR (400 MHz, D_2_O): 8.63 (s, 1H, C*H*), 7.41 (s, 1H, C*H*), 7.35 (s, 1H, C*H*), 4.15 (q, *J* = 7.3 Hz, 2H, C*H*_2_CH_3_), 3.82 (s, 3H, C*H*_3_), 1.94 (s, 3H, C*H*_3_CO_2_HF), 1.43 (t, *J* = 7.3 Hz, 3H, CH_2_C*H*_3_). ^19^F NMR (377 MHz, D_2_O): −128.87 (s, CH_3_CO_2_H*F*), −142.77 (m, *F*_2_H^−^), 27.1:1 ratio. H_2_O content: 1330 ppm.

1-Ethyl-3-methylimidazolium fluoride acetic acid, [EMIM]F.1.6AcOH (**6**.1.6AcOH)

Acetic acid (3.00 g, 50.0 mmol) was added to [EMIM]F.1.8EtOH (6.25 g, 29.2 mmol). The mixture was dried with an ethanol (50 mL) azeotrope, followed by drying for 5 h *in vacuo*. The product was a pale yellow liquid (6.50 g, 99%). ^1^H NMR (400 MHz, D_2_O): 8.63 (s, 1H, C*H*), 7.41 (s, 1H, C*H*), 7.35 (s, 1H, C*H*), 4.15 (q, *J* = 7.5 Hz, 2H, C*H*_2_CH_3_), 3.82 (s, 3H, C*H*_3_), 1.95 (s, 4.8H, C*H*_3_CO_2_HF), 1.42 (t, ^3^*J*_HH_ = 7.5 Hz, 3H, CH_2_C*H*_3_). ^19^F NMR (377 MHz, D_2_O): −128.88 (s, CH_3_CO_2_H*F*), −142.49 (m, *F*_2_H^−^), 24.0:1 ratio. H_2_O content: 1240 ppm.

1-Ethyl-3-methylimidazolium fluoride acetic acid, [EMIM]F.2.1AcOH (**6**.2.1AcOH)

Acetic acid (3.58 g, 59.6 mmol) was added to [EMIM]F.1.8EtOH (6.39 g, 29.8 mmol). The mixture was dried with ethanol (3 × 50 mL) azeotropes. Additional acetic acid (0.535 g, 8.91 mmol) was then added. The product was a pale yellow liquid (7.28 g, 96%). ^1^H NMR (400 MHz, D_2_O): 8.62 (s, 1H, C*H*), 7.40 (s, 1H, C*H*), 7.33 (s, 1H, C*H*), 4.14 (q, *J* = 7.3 Hz, 2H, C*H*_2_CH_3_), 3.80 (s, 3H, C*H*_3_), 1.95 (s, 6.3H, C*H*_3_CO_2_HF), 1.41 (t, *J* = 7.3 Hz, 3H, CH_2_C*H*_3_). ^19^F NMR (377 MHz, D_2_O): −128.81 (s, CH_3_CO_2_H*F*), −142.48 (m, *F*_2_H^−^), 28.4:1 ratio. H_2_O content: 1,030 ppm.

1-Ethyl-3-methylimidazolium fluoride acetic acid, [EMIM]F.2.4AcOH (**6**.2.4AcOH)

Acetic acid (4.09 g, 68.1 mmol) was added to [EMIM]F.1.8EtOH (5.83 g, 27.2 mmol). The mixture was dried with ethanol (2 × 50 mL) azeotropes. Additional acetic acid (0.495 g, 8.24 mmol) was then added. The product was a pale yellow liquid (7.48 g, 99%). ^1^H NMR (400 MHz, D_2_O): 8.60 (s, 1H, C*H*), 7.39 (s, 1H, C*H*), 7.32 (s, 1H, C*H*), 4.13 (q, ^3^*J*_HH_ = 7.3 Hz, 2H, C*H*_2_CH_3_), 3.79 (s, 3H, C*H*_3_), 1.95 (s, 7.2H, C*H*_3_CO_2_HF), 1.39 (t, ^3^*J*_HH_ = 7.3 Hz, 3H, CH_2_C*H*_3_). ^19^F NMR (377 MHz, D_2_O): −128.75 (s, CH_3_CO_2_H*F*), −142.46 (m, *F*_2_H^−^), 16.1:1 ratio. H_2_O content: 1,580 ppm.

1-Ethyl-3-methylimidazolium fluoride acetic acid, [EMIM]F.3.2AcOH (**6**.3.2AcOH)

Acetic acid (3.76 g, 62.6 mmol) was added to [EMIM]F.1.8EtOH (4.47 g, 20.8 mmol). The mixture was dried with ethanol (2 × 50 mL) azeotropes. Additional acetic acid (0.825 g, 13.7 mmol) was then added. The product was a pale yellow liquid (5.20 g, 77%). ^1^H NMR (400 MHz, D_2_O): 8.62 (s, 1H, C*H*), 7.40 (s, 1H, C*H*), 7.34 (s, 1H, C*H*), 4.14 (q, ^3^*J*_HH_ = 7.3 Hz, 2H, C*H*_2_CH_3_), 3.81 (s, 3H, C*H*_3_), 1.98 (s, 9.6H, C*H*_3_CO_2_HF), 1.42 (t, ^3^*J*_HH_ = 7.3 Hz, 3H, CH_2_C*H*_3_). ^19^F NMR (377 MHz, D_2_O): −128.80 (s, CH_3_CO_2_H*F*), −142.57 (m, *F*_2_H^−^), 20.4:1 ratio. H_2_O content: 900 ppm.

(2-hydroxyethyl)trimethylammonium fluoride hydrate, choline fluoride hydrate (**7**.H_2_O)

Choline chloride (10.2 g, 73.2 mmol) was dissolved into water (200 mL) in a blacked out flask. Ag_2_O (12.7 g, 54.8 mmol) was added and the solution stirred for 4 h. AgCl was removed by filtration through Celite and the filtrate was neutralized to pH 7.5 with aqueous HF. Water was removed by drying *in vacuo* at 40°C for 72 h to yield a white solid (9.89 g, 91%). Found: C, 40.34; H, 11.24; N, 9.03%. Calc. for C_5_H_14_NOF.1.43H_2_O: C, 40.34; H, 11.41; N, 9.41%.

## Results and Discussion

### Synthesis

**Scheme 1:**
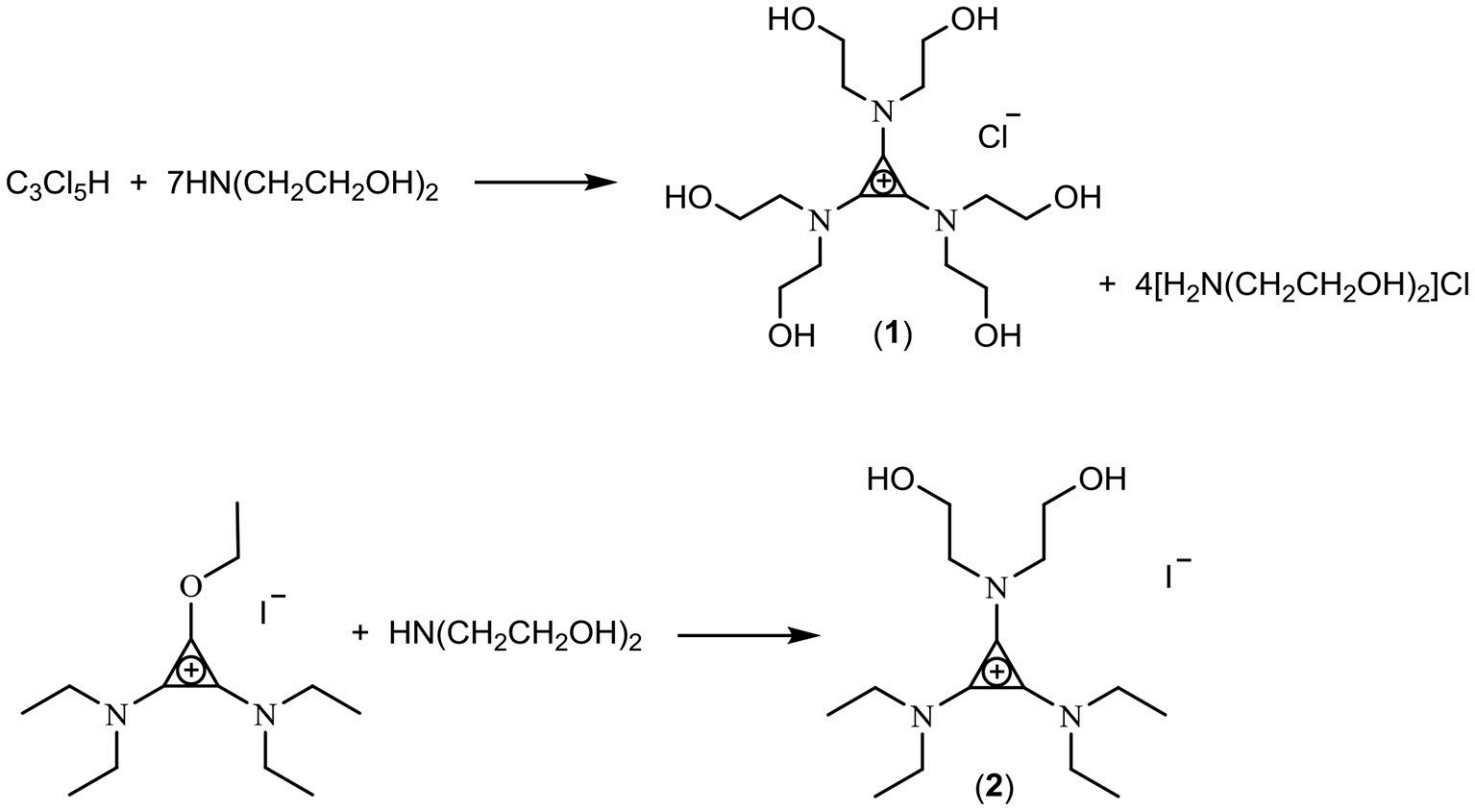
Synthesis of hydroxyl-functionalized TAC salts.

In this work, three classes of cations were studied for the preparation of fluoride ionic liquids: the tris(dialkylamino)cyclopropenium cation [C_3_(NPr_2_)_3_]^+^, the imidazolium cation [EMIM]^+^, and hydroxyl-functionalized salts. Two hydroxy-functionalized TAC salts were prepared: Treatment of C_3_Cl_5_H with HN(CH_2_CH_2_OH)_2_ yielded the hexaol TAC product [C_3_(N(CH_2_CH_2_OH)_2_)_3_]Cl (**1**) whereas a diol TAC salt was prepared by treatment of the alkoxydiamino TAC salt [C_3_(NEt_2_)_2_OEt]I (generated by addition of alkylation of the cyclopropenone (Et_2_N)_2_C_3_O with ethyliodide) with HN(CH_2_CH_2_OH)_2_ to give [C_3_(NEt_2_)_2_N(CH_2_CH_2_OH)_2_]I (**2**) (Scheme 1). Separation of the hexaol **1** from the excess starting amine and product ammonium salt proved challenging but was eventually achieved by trapping the smaller amine/ammonium salt in Zeolite 13X, which has a 10 Å pore size.

Three methods were investigated for the conversion of chloride salts to fluoride salts: anion metathesis with AgF, neutralization of hydroxide with HF acid, and metathesis with KF in ethanol.

Anion metathesis with AgF from the chloride salt is the most direct route (Maiti et al., 2008; Vitz et al., 2009): addition of AgF to an aqueous solution of a chloride IL precipitates AgCl. This method was used for one synthesis of [C_3_(N(C_2_H_4_OH)_2_)_3_]F (**3**) and resulted in a final chloride content of 1,600 ppm, ~3% of total anions.

A common and readily-accessible source of fluoride is HF. Since HF is a weak acid, direct metathesis with chloride is difficult, however, the chloride salt can be converted first to a hydroxide salt, which is then neutralized with HF. It should also be noted that TAC ILs are sensitive to concentrated or heated hydroxide solutions with the conversion of TAC cations to cyclopropenones. There were two methods trialed for the conversion of chloride to hydroxide anions: firstly, one using silver oxide and the other using sodium isopropoxide.

**Scheme 2:**
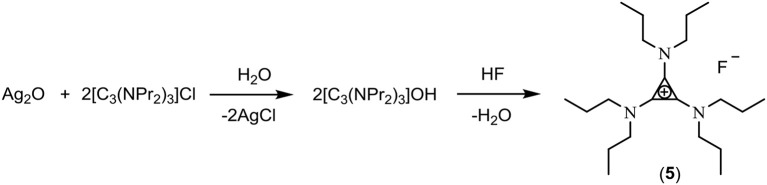
Exchange using silver oxide.

The exchange of chloride to hydroxide using Ag_2_O (Scheme 2) involves mixing Ag_2_O with an aqueous solution of the chloride salt in a blacked-out container. Using this method, chloride contents of 500 ppm were obtained. Solutions of **3**, [C_3_(NEt_2_)_2_N(C_2_H_4_OH)_2_]F (**4**) and [C_3_(NPr_2_)_3_]F (**5**) were synthesized using this method.

**Scheme 3:**
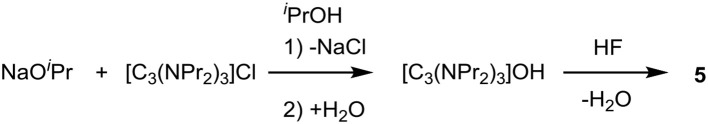
Exchange using sodium isopropoxide.

The exchange of chloride to hydroxide via isopropoxide (Scheme 3) involves mixing isopropanol solutions of sodium isopropoxide and the chloride salt, which precipitates NaCl, leaving the cations in solution with isopropoxide. When this isopropoxide salt is added to water, hydroxide anions are formed and neutralization with aqueous HF then generates the fluoride salt. In the case of **5**, the chloride content was 8%, which would significantly affect physical properties.

**Scheme 4:**
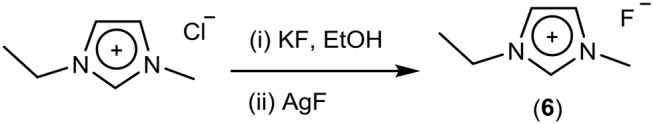
Synthesis of fluoride salts using KF in ethanol.

The final method used for the exchange of chloride to fluoride was the use of KF in ethanol (Scheme 4; Dermeik and Sasson, 1989). This method is similar to that of the sodium isopropoxide exchange of chloride to hydroxide. It involves partially dissolving potassium fluoride in ethanol, followed by the addition of the chloride salt. The solubility ratio of potassium fluoride to potassium chloride is 25, therefore, due to the lower solubility of potassium chloride, this precipitates out. The ethanol was then removed *in vacuo*, and acetonitrile was added. After cooling, a precipitate of potassium fluoride (and possibly residual chloride) was removed. Ethanol was added and acetonitrile removed *in vacuo* to yield the ethanol solvate. This method was used to synthesize the ethanol solvate of [EMIM]F (**6**), and after carrying out this exchange three times, the chloride content was 3,100 ppm, ~2.4% of total anions, and the potassium ion concentration was 220 ppm, ~0.15% of [EMIM]^+^. Due to the chloride concentration being higher than preferred, a further step of adding silver fluoride was carried out. The amount of silver fluoride that was added was calculated to lower chloride to < 100 ppm, without having excess silver present. The final chloride concentration was 70 ppm.

Once a solution of fluoride ionic liquid is obtained, the solvent needs to be removed, and this is a critical and delicate step. As the fluoride anion becomes less solvated, its basicity increases, which leads to decomposition. We will now discuss a variety of drying techniques that were trialed.

**Scheme 5:**
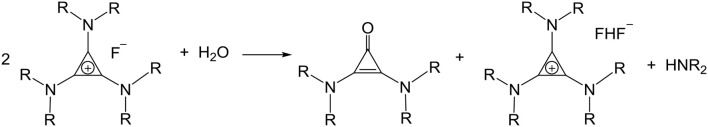
Decomposition of [C_3_(NPr_2_)_3_]F.

An aqueous solution of [C_3_(NPr_2_)_3_]F was dried on a rotary evaporator at 40°C. This reduced the water content to ~20%, about four water molecules per TAC fluoride. When the temperature or vacuum is increased more water is removed, however, due to the heat and the increasing basicity of the fluoride, the salt decomposed to bis(dipropylamino)cyclopropenone and [C_3_(NPr_2_)_3_]HF_2_ (Scheme 5). Freeze drying was also attempted, the water content dropped to 1.8% but there was 25% decomposition to cyclopropenone.

Heterogeneous azeotrope drying using toluene has been reported previously (Zhao et al., 2008), however, with the fluoride salts this still leads to the TAC fluoride salt being concentrated in a wet phase, and resulted in 30% cyclopropenone. Therefore, homogeneous azeotropes were investigated whereby water is removed before the ionic liquid is concentrated. Both ethanol and isopropanol will form lower boiling homogeneous azeotropes with water, and both were used successfully. Due to the potential of the ionic salt to break the azeotrope, a large excess of ethanol or isopropanol is used. Isopropanol has the advantage of having a higher percentage of water in the azeotrope, so water can be removed with less additional solvent.

Azeotrope drying was used to synthesize **3**, **4**.1.9EtOH, **5**.1.9EtOH, and **6**.1.8EtOH. During the synthesis of **5**.1.9EtOH, repeated additions of dry ethanol produced an ionic liquid fluoride with a water content of 2,200 ppm without cyclopropenone forming. The ethanol solvate could not easily be removed, because the strong hydrogen bonds between the alcohol solvate and fluoride anion provide stability to the IL fluoride.

Using another azeotrope, between benzene and ethanol, further ethanol can be removed from **5**.1.9EtOH to form a monoethanol solvate: one addition of dried benzene to a sample of the diethanol solvate leaves 1.36 ethanol molecules per fluoride. After two more additions, this drops to 1.1 ethanol molecule per fluoride. However, significant amounts (17%) of bis(dipropylamino)cyclopropenone were detected in this product.

A range of acetic acid solvates of an imidazolium fluoride were synthesized with ~1, 1.5, 2, 2.5, and 3 equivalents of acetic acid per fluoride anion. The acetic acid solvates were synthesized from the ethanol solvate **6**.1.8EtOH. Acetic acid was added and the mixture dried using ethanol or isopropanol azeotropes under reduced pressure with only small amounts of acetic acid being lost. Extra acetic acid was then added to give the desired HOAc/F^−^ ratio. ^1^H-NMR was used to determine the precise ratios of acetic acid to [EMIM]^+^, which were found to be 1.0, 1.6, 2.1, 2.4, and 3.2.

The ^13^C and ^19^F NMR spectra also provide evidence for the formation of fluoride hydrogen-bonded anions. The ^19^F NMR of **5**.1.9EtOH in D_2_O shows a peak at −122.03 ppm and **6**.1.8EtOH at −121.22 ppm, which is the shift for sodium fluoride in D_2_O. However, for the various acetic acid mixtures, the ^19^F NMR peak is around −128.8 ppm. This compares to the shift for [FHF]^−^, at −142 ppm, hence suggesting that [AcOHF]^−^ is persisting, or at least a significant contribution, in the D_2_O solution. A similar observation is made by examining the chemical shift of the methyl protons of the acetic acid. In D_2_O, acetic acid is observed at 2.08 ppm and the acetate ion at 1.90 ppm (Gottlieb et al., 1997), whereas for the fluoride acetic acid mixtures the chemical shifts are between 1.94 and 1.98 ppm. A small signal for [FHF]^−^ was also observed due to some dissociation of the fluoride solvate with the acetic acid then generating some H^+^, which can hydrogen bond to two fluorides.

### Properties

The viscosities of **5**.1.9EtOH and the [EMIM]F-acetic acid mixtures were measured over the temperature range 20–50°C (Table 1). Plots of viscosity and conductivity vs. temperature are provided in the Supplementary Information. The viscosity of **5**.1.9EtOH is lower than with DCA or ${\text{NTf}}_{2}^{-}$ anions (Walst et al., 2015) whereas the [EMIM]F acetic acid solvates are similar to the [EMIM]DCA salt. This is due to the acetic acid or ethanol being a co-solvent with much lower viscosity. While it is not possible to directly compare the viscosities of the acetic acid and ethanol solvates, due to the different cations and different number of solvate molecules, it is seems likely that the viscosity of the ethanol solvates is lower than the acetic acid solvates if the cation and number of solvate molecules is the same. Note that the viscosity of [C_3_(NPr_2_)_3_]NTf_2_ (Table 1) is approximately five times greater than that of [EMIM]NTf_2_ (Walst et al., 2015), so an increase by a factor of about 2.2 from the EMIM acetic acid solvates to **5**.EtOH is small.

**Table 1:** Viscosity data for fluoride solvates (mPa s).

**Salt**	**Temperature (°C)**						
	**20**	**25**	**30**	**35**	**40**	**45**	**50**
**5**.1.9EtOH	49.6	40.3	32.7	26.8	22.1	18.1	15.4
[C_3_(NPr_2_]_3_]NTf_2_	220		127		78.3		50.1
[C_3_(NPr_2_]_3_]DCA	107		59		35		21
**6**.1.0AcOH	30.2	24.7	20.2	17.2	14.7	12.7	11.1
**6**.1.6AcOH	20.5	17.1	14.8	12.7	11.1	9.71	8.58
**6**.2.1AcOH	17.6	14.8	12.7	11.0	9.61	8.45	7.47
**6**.2.4AcOH	15.9	13.4	11.5	9.92	8.65	7.60	6.71
**6**.3.2AcOH	13.8	11.8	10.1	8.82	7.73	6.81	6.03
[EMIM]DCA	19.1	16.1	14.0	11.9	10.4	9.1	8.2
[EMIM]NTf_2_	38.6		27.1		19.4		14.9

The temperature dependence (Table 2) was modeled with the VFT and Arrhenius equations (see Supplementary Information). D values (*B*/*T*_0_) of between 12.1 and 14.3 were found for the acetic acid solvates and 10.1 for **5**.1.9EtOH, suggesting that these solvates are slightly less fragile than other ionic liquids with the same cations. The activation energies are between 22 and 31 kJ mol^−1^.

**Table 2:** Fitting parameters for temperature dependence of viscosity.

**Salt**	**η_**0**_** **mPa s**	**B** **K**	***T*_**0**_** **K**	**D**	**δ** **mPa s**	***A* × 10^**−3**^** **mPa s**	***E*_**a**_** **kJ mol^**−1**^**	**δ** **mPa s**
**5**.1.9EtOH	0.020	1292	128	10.1	0.108	0.15	31	0.12
**6**.1.0AcOH	0.029	1290	106	12.1	0.024	0.60	26	0.28
**6**.1.6AcOH	0.030	1289	95	13.5	0.030	1.81	23	0.16
**6**.2.1AcOH	0.028	1289	93	13.9	0.015	1.76	22	0.12
**6**.2.4AcOH	0.024	1289	94	13.8	0.007	1.48	23	0.10
**6**.3.2AcOH	0.024	1289	90	14.3	0.004	1.85	22	0.06

Addition of acetic acid decreases the viscosity, for example, from 30.2 with 1.0AcOH to 13.8 mPa s with 3.2AcOH at 20°C. This is due to the acetic acid being a lower viscosity liquid and indicates the presence of increasing amounts of free HOAc.

The conductivities were measured over a temperature range of 20–40°C (Table 3). The conductivity of **5**.1.9EtOH is higher than the corresponding ${\text{NTf}}_{2}^{-}$ salt whereas the [EMIM]F acetic acid solvates have lower conductivity than the [EMIM]DCA ionic liquid. This is a reflection of the trends seen with the viscosity measurements. As with viscosity, it is not possible to directly compare their viscosities of the acetic acid and ethanol solvates, due to the different cations and different number of solvate molecules, it is probable that the conductivity of ethanol solvates is higher than the acetic acid solvates if the cation and number of solvate molecules is the same. The conductivity of the ethanol solvate is lower due to the large [C_3_(NPr_2_)_3_]^+^ cation, which causes higher viscosity and lowers the number of charge carriers in a given volume.

**Table 3:** Conductivity data and fitting parameters for fluoride solvates (mS cm^−1^).

**Salt**	**Temperature (°C)**					***A* × 10^**4**^**	***E*_**a**_**	**δ**
	**20**	**25**	**30**	**35**	**40**	**mS cm^**−1**^**	**kJ mol^**−1**^**	**mS cm^**−1**^**
**5**.1.9EtOH	1.89	2.28	2.74	3.24	3.75	9.19	26	0.03
[C_3_(NPr_2_)_3_]NTf_2_	0.50		0.86		1.32			
**6**.1.0AcOH	9.37	10.8	12.4	14.3	16.2	5.16	21	0.03
**6**.1.6AcOH	11.7	13.2	14.8	17.0	19.1	2.81	19	0.12
**6**.2.1AcOH	10.8	12.5	14.3	16.2	18.6	3.86	20	0.07
**6**.2.4AcOH	10.6	11.9	13.4	14.9	16.6	1.24	17	0.01
**6**.3.2AcOH	10.0	11.5	13.0	14.7	16.4	2.25	19	0.05
[EMIM]DCA	27							
[EMIM]NTf_2_	7.73	9.12		12.29				

The temperature dependence of the conductivity (Table 3) was modeled with the Arrhenius equation (see Supplementary Information). The activation energies were between 17 and 26 kJ mol^−1^. Due to the very limited temperature range and limited number of data points, the fitting to the VFT equation did not produce sensible results, so they are not reported here.

**Figure 1:**
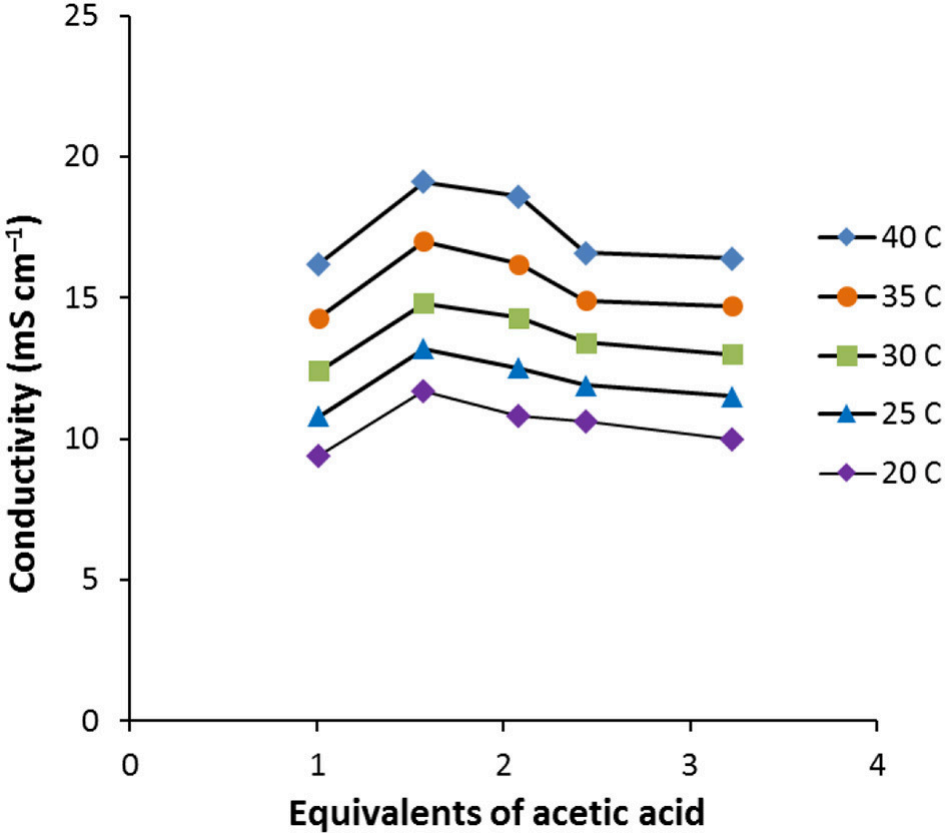
Conductivity vs. equivalents of acetic acid for **6**.nAcOH (*n* = 1.0, 1.6, 2.1, 2.4, 3.2).

As the amount of acetic acid (Figure 1) increases, the conductivity increases from 9.4 mS cm^−1^ for [C_2_MIM]F.1.0AcOH to 11.7 mS cm^−1^ for [C_2_MIM]F.1.6AcOH, it then decreases to 10.0 mS cm^−1^ for [C_2_MIM]F.3.2AcOH as further acetic acid is added. Initially, the molar conductivity increases, consistent with decreasing viscosity, but further acetic acid decreases the concentration of ions.

The densities for the [EMIM]F acetic acid mixtures were measured from 20 to 50°C (Table 4). As expected, density decreases as the relative concentration of acetic acid increases due to the lower density of acetic acid, and the density is linearly dependent on temperature (Table 5).

**Table 4:** Density data for imidazolium fluoride solvates (g mL^−1^).

**Salt**	**Temperature (°C)**						
	**20**	**25**	**30**	**35**	**40**	**45**	**50**
**6**.1.0AcOH	1.125	1.122	1.118	1.115	1.112	1.108	1.105
**6**.1.6AcOH	1.121	1.117	1.114	1.110	1.107	1.103	1.100
**6**.2.1AcOH	1.120	1.116	1.112	1.109	1.105	1.101	1.098
**6**.2.4AcOH^a^	1.119	1.115	1.111	1.107	1.104	1.100	1.096
**6**.3.2AcOH	1.118	1.114	1.110	1.106	1.102	1.098	1.094

a *By interpolation*.

**Table 5:** Fitting parameters for temperature dependence of density, ρ *=* *a – bT*.

**Salt**	***b* × 10^**4**^** **g mL^**−1**^ K^**−1**^**	***a*** **g mL^**−1**^**	**δ × 10^**5**^**
**6**.1.0AcOH	6.7834	1.3238	2.5
**6**.1.6AcOH	7.0611	1.3276	2.6
**6**.2.1AcOH	7.2967	1.3335	2.6
**6**.3.2AcOH	7.6728	1.3424	2.8

**Figure 2:**
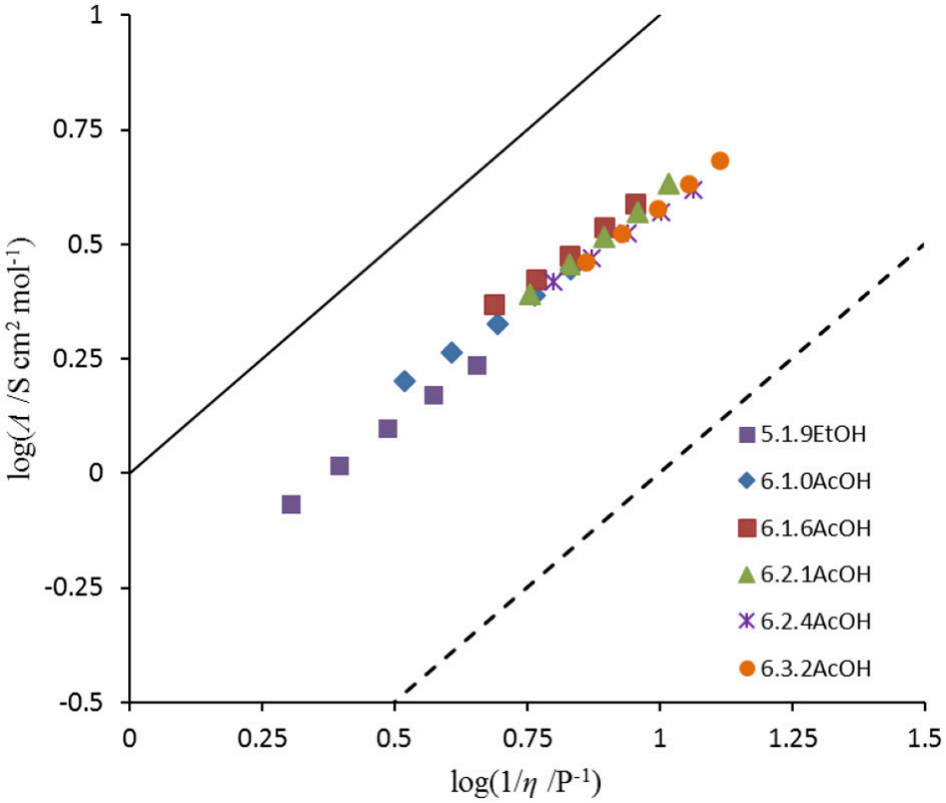
Walden plot of fluoride solvates.

The ionicity of the fluoride solvates was assessed using a Walden plot (Figure 2; Xu et al., 2003). To complete the set of calculations, the density of **5**.1.9EtOH was approximated by that of [C_3_(NPr_2_)_3_]DCA. The Walden product (= σ.η) and the deviation from ideal ionic behavior in the Walden plot, Δ*W*, are given in Table 6. The Walden products for the fluoride solvates are between 0.40 and 0.48 S cm^2^ P mol^−1^ and deviations from the ideal ionic behavior are between 0.32 and 0.40. Hence, all of the fluoride solvate ionic liquids can be classified as good ionic liquids in terms of the ionicity (Xu et al., 2003), with some degree of ion association or correlations evident suggesting some residual specific interaction between the cation and the anion solvate. There is a slight increase in Δ*W* as the amount of acetic acid increases. As a comparison, **6**.2.3HF has a higher Walden product of 0.76 and a lower Δ*W* of 0.12 (Hagiwara et al., 2003); in that case, there is the potential for a contribution from proton hopping, which increases molar conductivity.

**Table 6:** Walden product and deviation from ideal at 20°C.

**Salt**	**Walden product**	**Δ*W***
**5**.1.9EtOH	0.43	0.37
**6**.1.0AcOH	0.48	0.32
**6**.1.6AcOH	0.48	0.32
**6**.2.1AcOH	0.43	0.36
**6**.2.4AcOH	0.42	0.38
**6**.3.2AcOH	0.40	0.40

**Figure 3:**
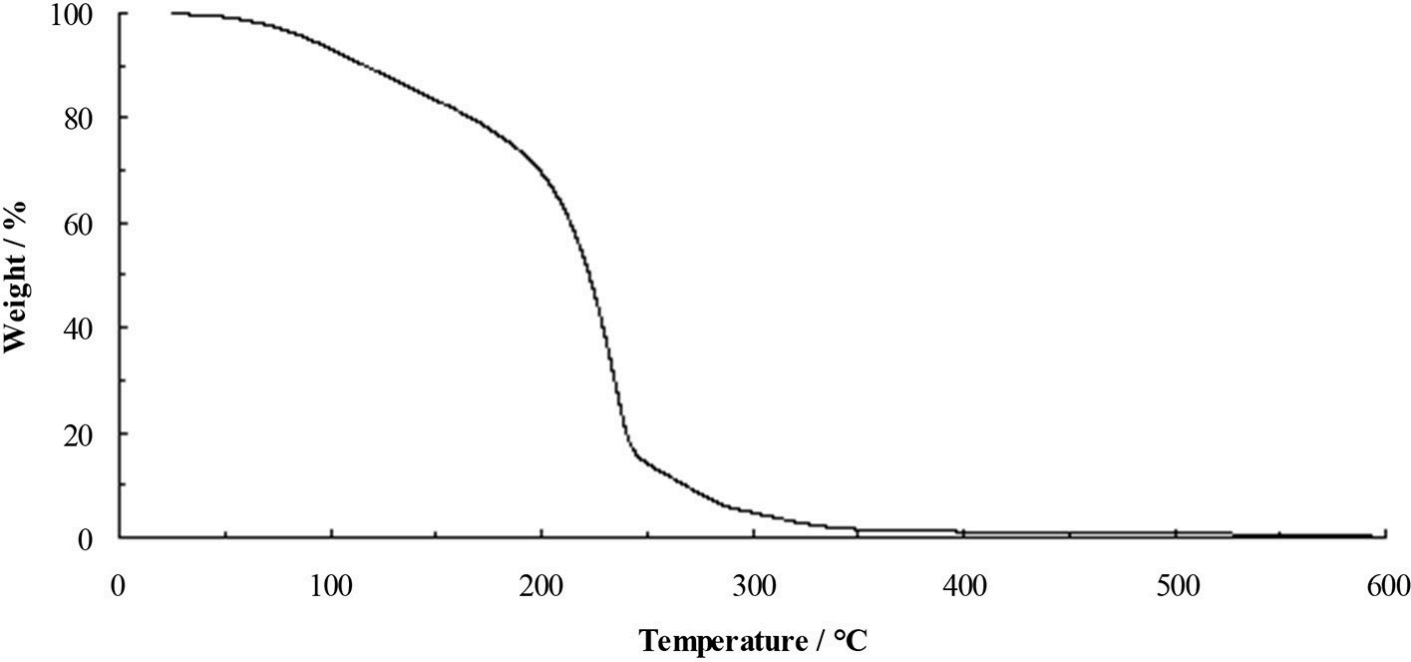
TGA of 5.1.9EtOH at 10°C min^−1^.

Thermally stable ionic liquids would be desirable as they could be used in applications at elevated temperatures, however, the reactivity of fluoride and the volatility of acetic acid and ethanol are expected to hinder stability. The TGA of the TAC fluoride ethanol solvate **5**.1.9EtOH (Figure 3) shows steady weight loss from ~50°C. Initially, this is likely to be loss of ethanol, which will lead to the decomposition of the cation.

**Figure 4:**
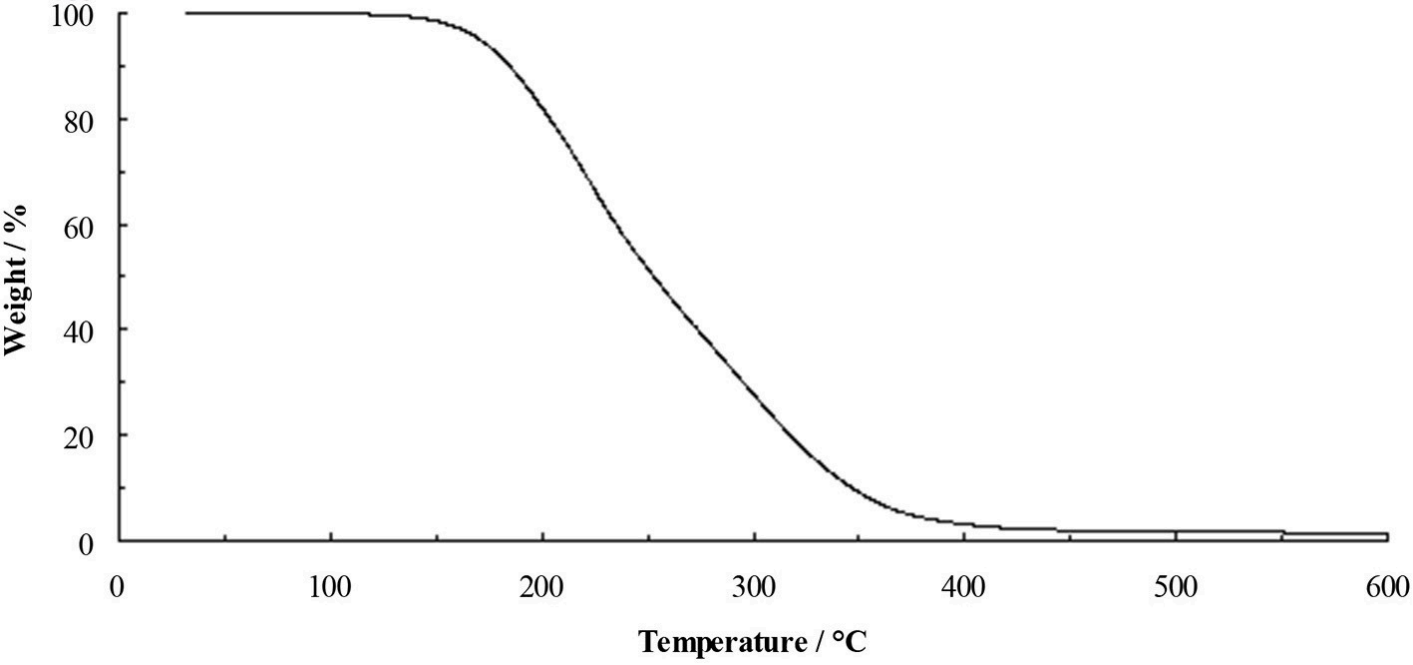
TGA of [C_3_(NEt_2_)_2_N(C_2_H_4_OH)_2_]F **(4)** at 10°C min^−1^.

The TGA of the dihydroxyl derivative **4** (Figure 4) shows a profile similar to that of [C_3_(N(C_2_H_4_OH)_2_)_3_]F, with little weight loss before onset of decomposition at 175°C. This clearly indicates that hydroxyl functional groups do stabilize the fluoride anion. Salt **4** is a highly-viscous yellow liquid at ambient temperature, however, it is very sensitive to trace water and much care needs to be taken when drying this material.

The TGA of the hexahydroxyl derivative **3** shows an increase in stability compared to **4** with onset of decomposition at 191°C (Supplementary Information). The onset temperature is, as expected, much lower than other ionic liquids with non-coordinating anions such as ${\text{NTf}}_{2}^{-}$, which can exceed 400°C. Potentially useful is the observation of the melting point at 87°C, which indicates a stable ionic liquid range at elevated temperatures. It is notably less sensitive to moisture during the drying process compared to **4**. A sample of (2-hydroxyethyl)trimethylammonium (choline) fluoride hydrate (**7**.H_2_O) was prepared from the chloride salt using silver oxide and aqueous HF. The TGA shows good thermal stability compared to the ethanol solvate of **5**, with an onset temperature of 139°C (Supplementary Information). However, this salt is a solid and no melting point is observed prior to decomposition.

The thermal behavior of **6**.1.6AcOH and **6**.3.2AcOH were examined using DSC: glass transitions were observed at −92 and −95°C, respectively, however, no melting points were observed.

## Conclusions

Ionic liquid fluorides were synthesized by exchange of chloride to fluoride using one of three methods: anion metathesis with silver fluoride, anion metathesis using aqueous silver oxide followed by neutralization with HF acid or anion metathesis with potassium fluoride in ethanol. The fluoride salts were then isolated using homogeneous azetrope drying.

Whereas, naked fluoride salts are not stable in pure form, fluoride solvates with ethanol and acetic acid were found to be much more stable, probably due to strong hydrogen bond formation. Increasing amounts of acetic acid solvate decreases the viscosity and density, while the conductivity peaked at **6**.1.6AcOH.

The low decomposition temperature of **5**.1.9EtOH (starting at 50°C) led to the rational design of ionic liquids containing hydroxyl-functionalized cations; **3** and **4**. These liquids demonstrate improved thermal stability with higher decomposition temperatures of 175 and 191°C, respectively, consistent with stabilization by intramolecular hydroxyl-fluoride bonding, leading to ionic liquids that are stable as the unsolvated ionic liquid fluorides.

## Author Contributions

OC conceived of the project and directed the TAC work. DM conceived of the acetic acid solvate concept and directed this work. KW carried out the experimental work. All authors made critical contributions to the report, gave significant input into the report writing process and analysis of data herein.

### Conflict of Interest Statement

The authors declare that the research was conducted in the absence of any commercial or financial relationships that could be construed as a potential conflict of interest.
