# Supplementary material for: Fluoride Ionic Liquids in Salts of Ethylmethylimidazolium and Substituted Cyclopropenium Cation Families
Source: Front Chem. 2018 Dec 10;6:603. doi: 10.3389/fchem.2018.00603 (PMC6295474; doi:10.3389/fchem.2018.00603)
Supplement: Supplementary file 1 [file Data_Sheet_1.doc]

**Supplementary Information**

**Fluoride Ionic Liquids in Salts of Ethylmethylimidazolium and Substituted Cyclopropenium Cation Families**

### *Owen J. Curnow^1^*, Douglas R. MacFarlane^2^* and Kelvin J. Walst^1^*

*^1^ School of Physical and Chemical Sciences, Sciences, University of Canterbury, Private Bag 4800, Christchurch 8041, New Zealand, ^2^ ARC Centre of Excellence for Electromaterials Science, Monash University, Victoria 3800, Australia*

**Fitting of the viscosity data using the Arrhenius and VFT equations**

The viscosity data was fit to both the Arrhenius equation (*η* = *A*exp(*E*_a_/R*T*)) and the Vogel-Fulcher-Tammann (VFT) equation (also note that *D* = *B*/*T*_0_):


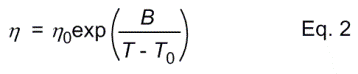


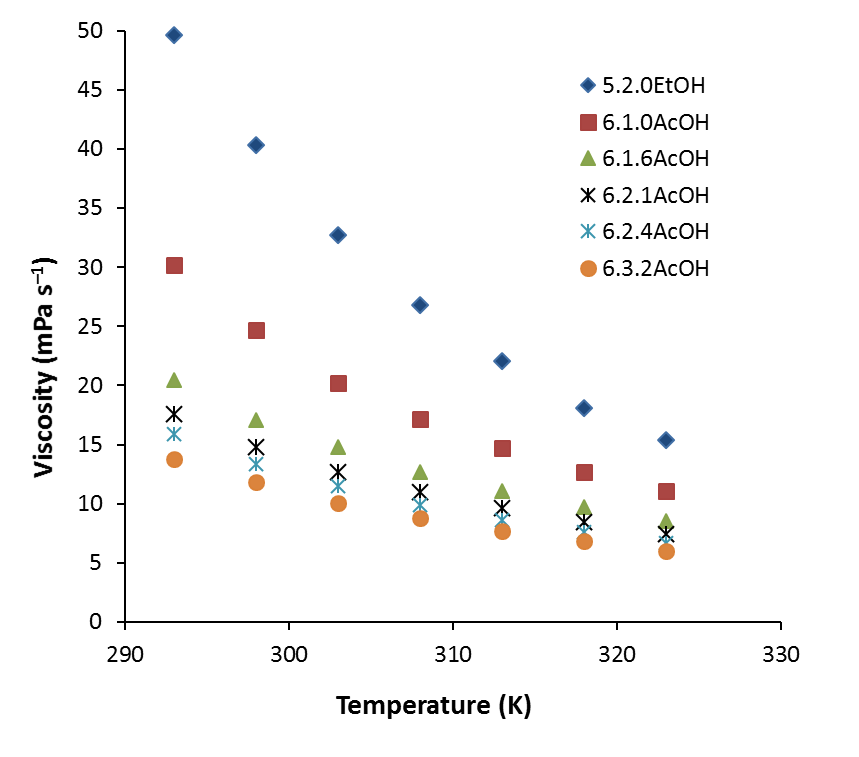


**Figure 1S.** Viscosity versus temperature for ionic liquid fluoride solvates of **5** and **6**.

The conductivity data was fit to only the Arrhenius equation (*σ* = *A*exp(–*E*_a_/R*T*)).

**
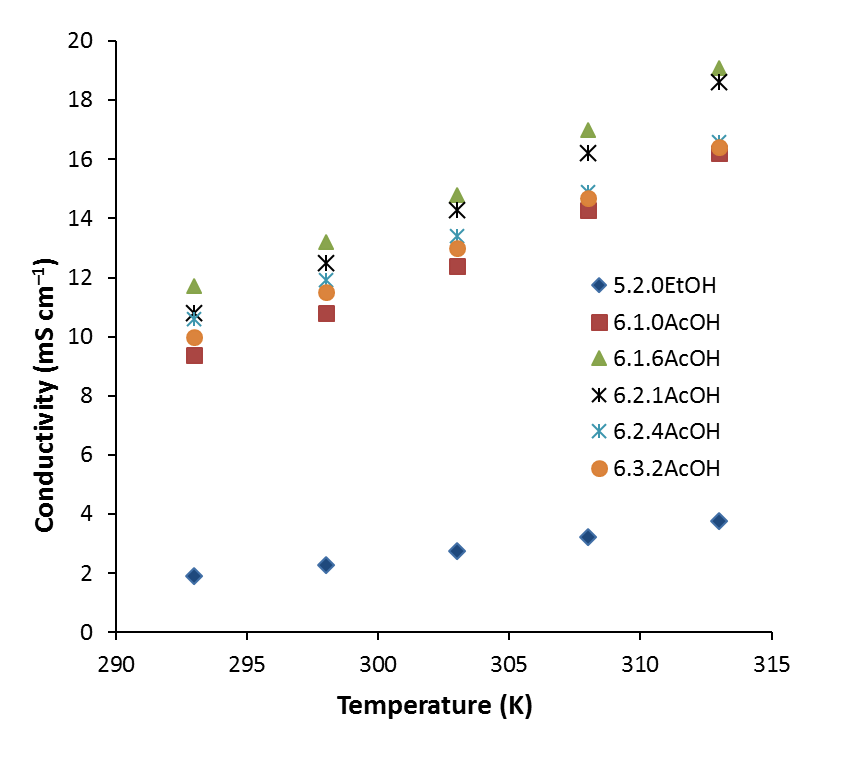
**

**Figure 2S.** Conductivity versus temperature for ionic liquid fluoride solvates of **5** and **6**.

**Figure 3S.** TGA of [C_3_(N(C_2_H_4_OH)_2_)_3_]F (**3**) at 10 °C min^–1^.

**Figure 4S.** TGA of (2-hydroxyethyl)trimethylammonium fluoride hydrate (**7**.H_2_O) at 10 °C min^–1^.

**
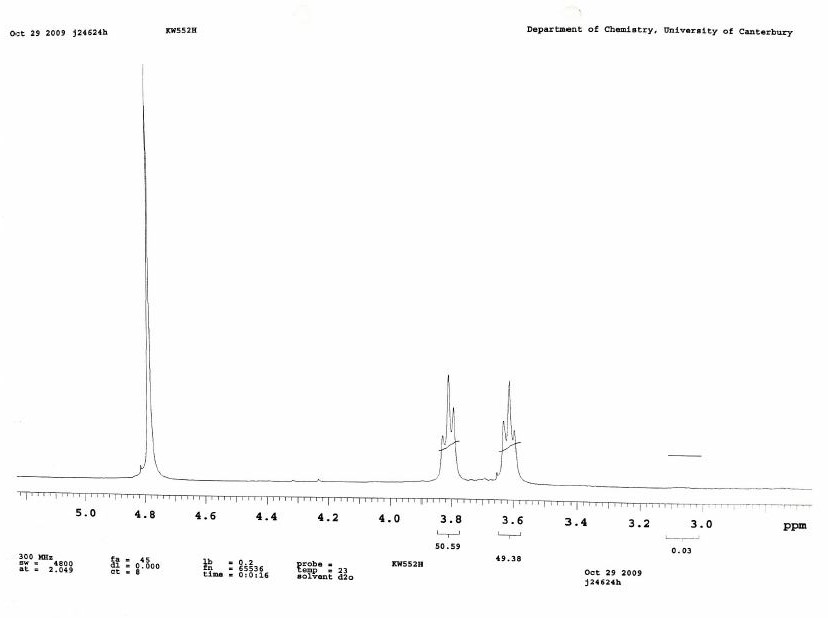
**

**Figure 5S.** ^1^H-NMR spectrum of [C_3_(N(CH_2_CH_2_OH)_2_)_3_]Cl (**1**) in D_2_O.


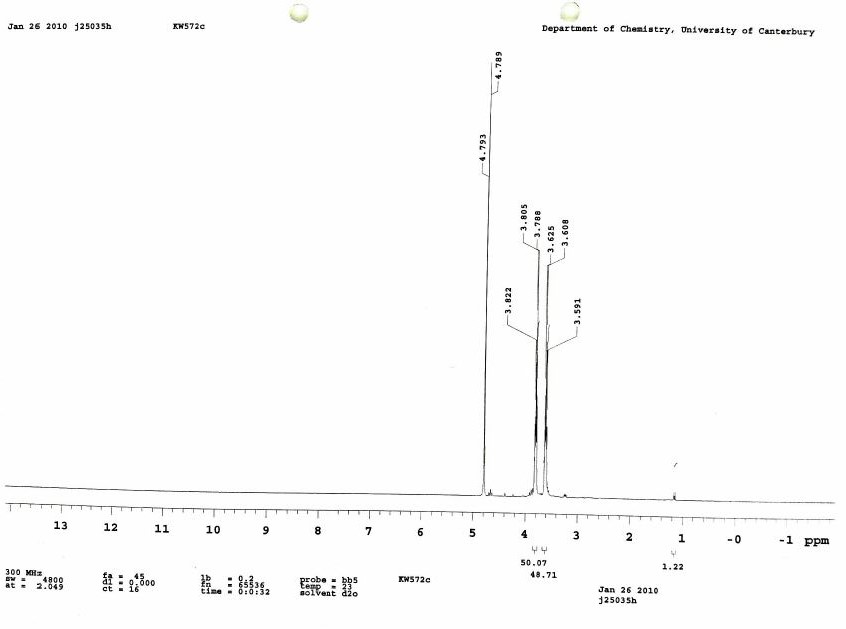


**Figure 6S.** ^1^H-NMR spectrum of [C_3_(N(CH_2_CH_2_OH)_2_)_3_]F (**3**) in D_2_O.


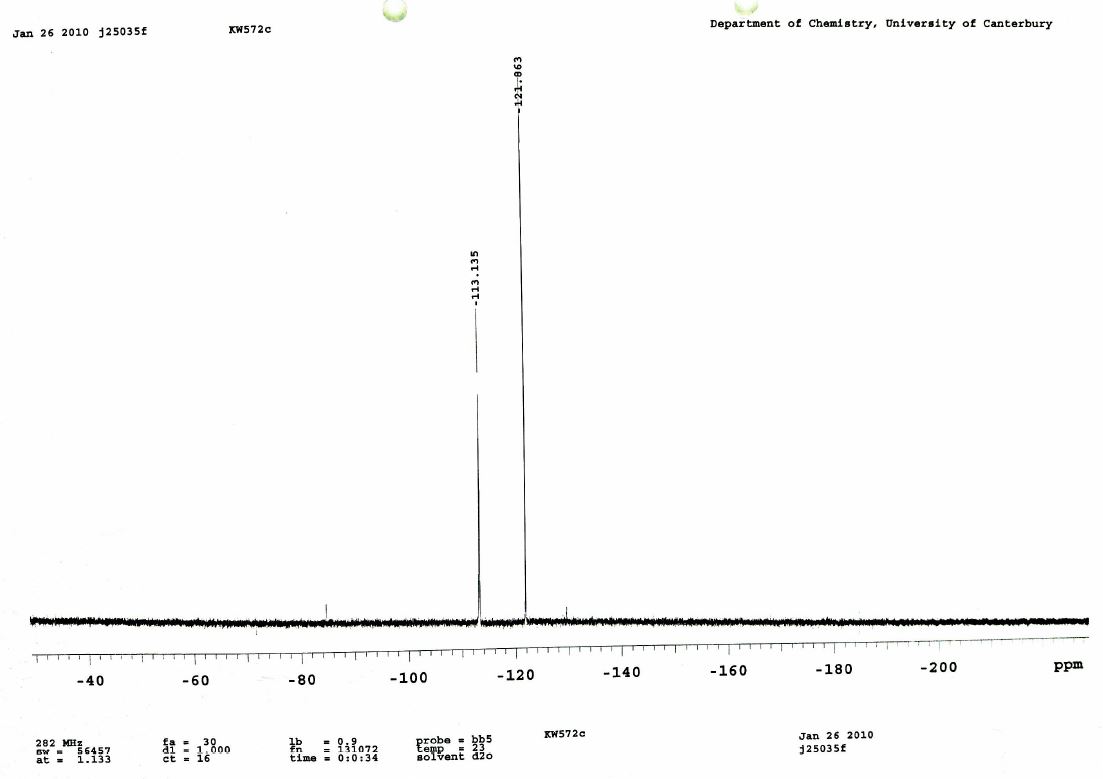


**Figure 7S.** ^19^F-NMR spectrum of [C_3_(N(CH_2_CH_2_OH)_2_)_3_]F (**3**) in D_2_O.


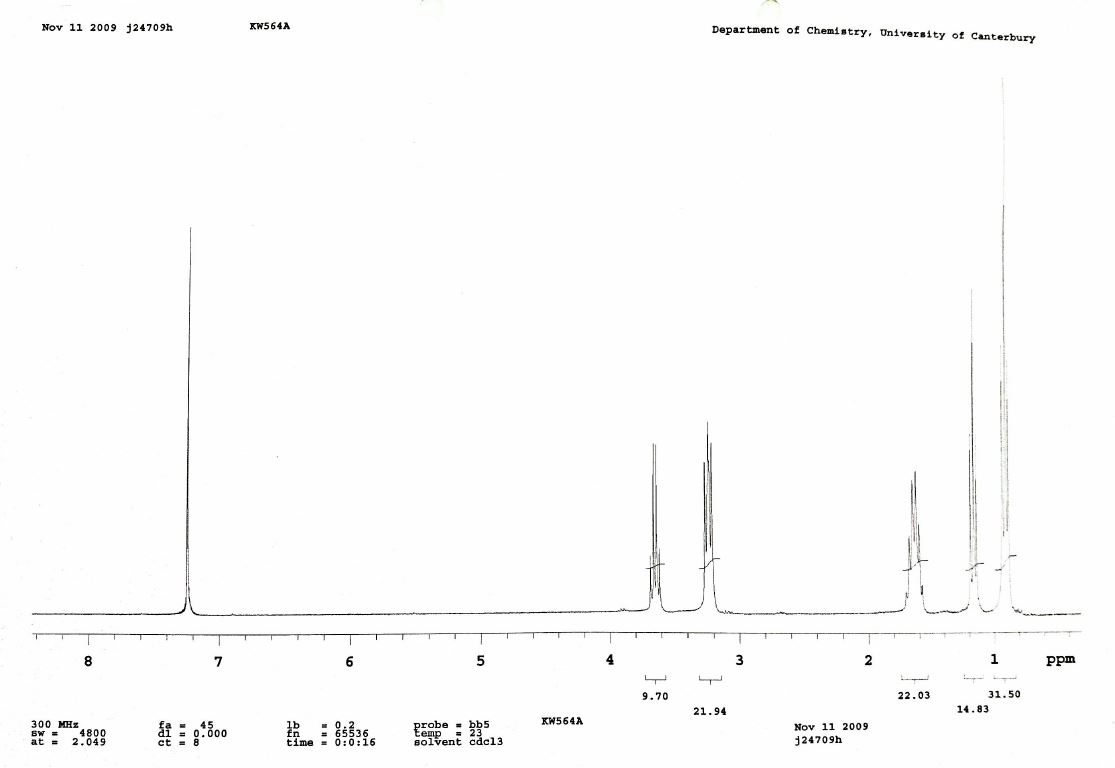


**Figure 8S.** ^1^H-NMR spectrum of [C_3_(NPr_2_)_3_]F.3EtOH (**5.**3EtOH) in CDCl_3_.


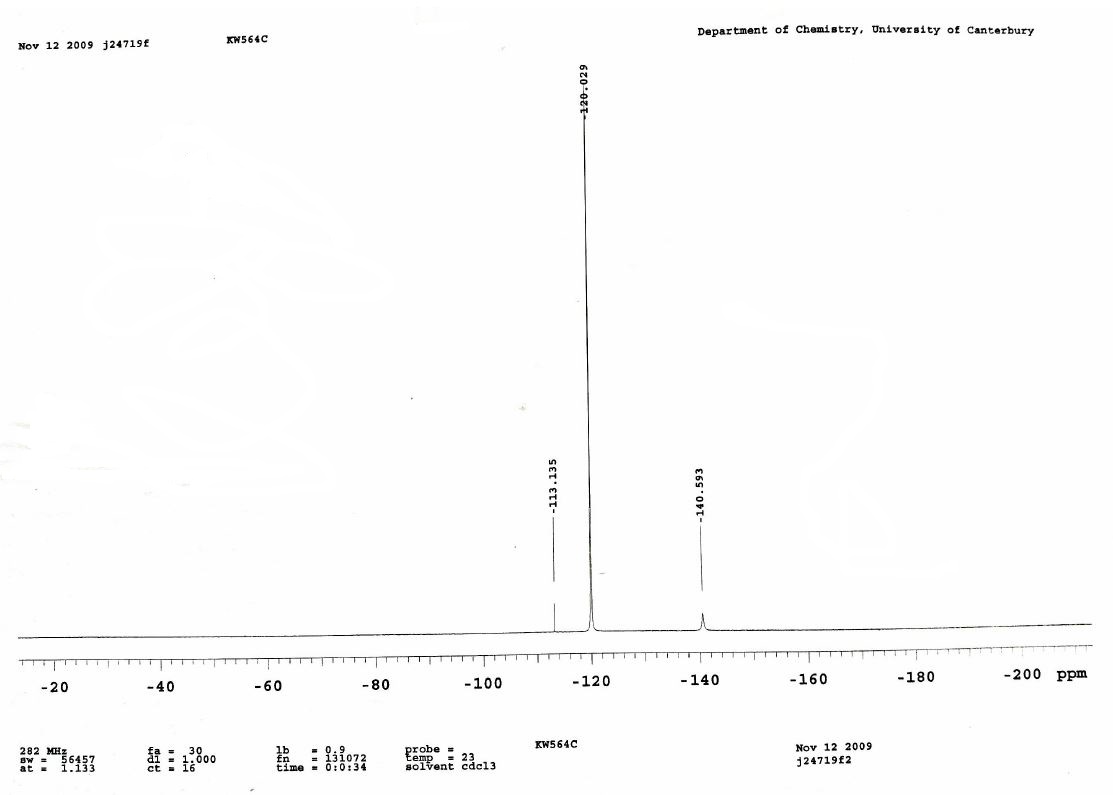


**Figure 9S.** ^19^F-NMR spectrum of [C_3_(NPr_2_)_3_]F.3EtOH (**5.**3EtOH) in CDCl_3_.
